# Stimulation of prostate cells by the senescence phenotype of epithelial and stromal cells: Implication for benign prostate hyperplasia

**DOI:** 10.1096/fba.2018-00084

**Published:** 2019-04-09

**Authors:** Shoulei Jiang, Chung Seog Song, Bandana Chatterjee

**Affiliations:** ^1^ Department of Molecular Medicine University of Texas Health San Antonio San Antonio Texas; ^2^ South Texas Veterans Health Care System San Antonio Texas; ^3^ Present address: Department of Medicine University of Texas Health San Antonio San Antonio Texas

**Keywords:** AKT, BPH, ERK1/2, inflammation, SASP, STAT5

## Abstract

Hyperproliferation of prostate transition‐zone epithelial and stromal cells leads to benign prostate hyperplasia (BPH), a prevalent pathology in elderly men. Senescent cells in BPH tissue induce a senescence‐associated secretory phenotype (SASP) which, by generating inflamed microenvironment and reactive stroma, promotes leukocyte infiltration, cellular hyperproliferation, and nodular prostate growth. We examined human prostate epithelial (BPH‐1, PNT‐1α) and stromal (HPS‐19I) cells for SASP induction by ionizing radiation and assessed SASP's impacts on cell proliferation and on signal transducers that promote cellular growth, proliferation, and survival. Radiation‐induced DNA damage led to cellular senescence, evident from elevated expression of senescence‐associated β‐galactosidase and the cell‐cycle inhibitor p16/INK4a. Clinical BPH tissue showed p16 accumulation. SASP induced mRNA expression for inflammatory cytokines (IL‐1α, IL‐6, IL‐8, TNF‐α); chemokines (GM‐CSF, CXCL12); metalloproteases (MMP‐1, MMP‐3, MMP‐10); growth factor binding IGFBP‐3. Media from irradiated epithelial or stromal cells enhanced BPH‐1 proliferation. ERK1/2 and AKT, which enhance cell growth/survival and STAT5, which facilitates cell cycle progression and leukocyte recruitment to epithelial microenvironment, were activated by SASP components. The radiation‐induced cellular senescence model can be a platform for identification of individual SASP components and pathways that drive BPH etiology/progression in vivo and targeting them may form the basis for novel BPH therapy.

**Abbreviations**
AR: androgen receptor
BPH: benign prostate hyperplasia
CDK: cyclin‐dependent kinase
CXCL: CXC‐motif chemokine ligand
ELISA: enzyme‐linked immunosorbent assay
ERK: extracellular signal‐related kinase
GM‐CSF: granulocyte‐macrophage colony‐stimulating factor
HPS: human prostate stroma
IGFBP: insulin growth factor binding protein
IHC: immunohistochemistry
IL: interleukin
IR: ionizing radiation
LHRH: luteinizing‐hormone releasing hormone
LUTS: lower urinary tract symptoms
MMP: metalloprotease
PCR: polymerase chain reaction
SASP: senescence‐associated secretory phenotype
SA‐βGal: senescence‐associated β‐galactosidase
STAT: signal transducer and activator of transcription

## 1 INTRODUCTION

Hyperproliferation of non‐tumorigenic prostate cells, especially prostate epithelial cells, leads to benign prostate hyperplasia (BPH), a progressive disease that affects about ~50% men in their 50s and 60s, and up to 80% men by age 85.^1^, ^2^, ^3^ BPH manifests as new glandular epithelial growth and, less frequently, stromal cell growth at the prostate transition zone surrounding the upper portion of the prostatic urethra—a site less susceptible to cancer development. Androgen action via androgen receptor signaling is closely linked to BPH,^4^ which is in keeping with the efficacy of 5‐α reductase inhibitors in alleviating BPH‐associated lower urinary tract symptoms (LUTS). Inhibition of 5‐α reductase blocks testosterone conversion to the reduced metabolite, that is, 5α‐dihydrotestosterone, the mediator of androgen action in the prostate. Blockers of the α1‐adrenergic receptor that decrease smooth muscle tension in the prostate stroma, urethra, and bladder neck, also provide relief from BPH.^5^ Disadvantages of anti‐androgen/anti‐α1‐receptor therapy include a lack of universal drug response and side effects ranging from sexual adversity to loss of control of the lower urinary tract muscle leading to voiding problems. Epithelial and stromal factors that influence the interplay between aging and prostate cell growth may inform new avenues for the optimal intervention of BPH/LUTS.

Benign prostate hyperplasia epithelium is enriched for senescent cells.^6^ Cellular senescence is known to occur in the prostate and various other human tissues during normal aging, in progeric patients manifesting accelerated aging, and in the course of replicative exhaustion of passaged cell populations. Genotoxic stress from DNA damage or oncogene activation also evokes cellular senescence.^7^, ^8^, ^9^ Senescent cells are metabolically active, but irreversibly arrested at the G1/S cell cycle check point. As a cell‐intrinsic tumor‐suppressive mechanism, cellular senescence bars aberrant proliferation of cells that are irreversibly damaged by insults such as oxidative stress induced by cell's metabolic activities, chromosomal instability from shortened telomeres, and DNA damage by chemicals or ionizing radiation. Elevated expression of the p16^INK4a^/CDKN2a tumor suppressor is associated with cellular senescence in many tissues. By inhibiting cyclin‐dependent kinases CDK4/6 and CDK2, p16/INK4a helps maintain the retinoblastoma protein in a hypo‐phosphorylated state, which leads to the sequestration of E2F transcription factors and thus G1/S arrest due to inhibition of the E2F‐dependent expression of DNA synthesis genes. Clearance of p16‐positive senescent cells delayed age‐associated functional decline in a mouse model.^10^ Senescence‐associated β‐galactosidase (SA‐βGal) activity at pH 6.0 is another marker for senescent cells.^7^ Cells of the BPH epithelium and late‐passage epithelial cells isolated from the normal prostate transition zone showed elevated p16/INK4a and SA‐βGal.^6^, ^11^, ^12^ The tumor‐suppressive potential of senescent cells is mitigated in some contexts by a senescence‐associated secretory phenotype (SASP). By secreting inflammatory cytokines, chemokines, growth factors, and proteases, an SASP‐stimulated tissue microenvironment promotes infiltration of inflammatory cells which, by sparking growth stimulation of neighboring cells, can initiate errant tissue growth.^7^, ^13^

This study shows that epithelial and stromal cells of the normal human prostate developed SASP upon exposure to ionizing radiation (IR) in cell culture, and conditioned media from SASP‐induced cells stimulated proliferation of non‐irradiated prostatic epithelial cells. SASP was associated with (a) elevated mRNAs for inflammatory cytokines, chemokines, growth factors, and metalloproteases; (b) activation of the signal transducer STAT5, which promotes cell cycle progression and leukocyte infiltration into tissue microenvironment; and (c) activation of ERK1/2 and AKT, which regulate cell growth and survival. Irradiated prostate cells and resulting premature senescence can be used as an experimental platform to identify SASP components and signal networks that regulate BPH pathogenesis, and their targeting may afford novel intervention of this disease.

## 2 MATERIALS AND METHODS

### 2.1 Cell lines, culture conditions, cell irradiation

Immortalized, non‐malignant prostate epithelial cell lines BPH‐1^14^ (ATCC, Manassas, VA) and PNT‐1α (European Collection of Cell Culture, Porton Down, UK) were used. BPH‐1 originated from a human BPH prostate specimen; PNT‐1α is a clonal line of the human PNT‐1 line, which was generated from prostate epithelial cells of a normal adult.^15^ HPS‐19I primary prostate stromal cells were grown out of the prostate stroma of a 19‐year‐old male.^16^, ^17^ HPS‐19I cells are vimentin+ and smooth muscle actin‐negative, indicating a fibroblast‐like phenotype. BPH‐1 and PNT‐1α were cultured in RPMI‐1640 and 10% (v/v) fetal bovine serum (FBS, GEMINI Bio‐products, West Sacramento, CA) plus penicillin (100U/ml) and streptomycin (100 µg/mL). HPS‐19I was cultured in DMEM‐HG (Gibco) containing 5% FBS(v/v), 5% Nu‐serum (v/v, Corning #355504), insulin (5 µg/mL), testosterone (0.5 µg/mL), and penicillin/streptomycin. Cells were maintained in a humidified incubator at 37**°**C and 5% CO_2_.

Cell lines were mycoplasma free and ensured for authenticity based on source (HPS19I from Dr David Rowley, Baylor College of Medicine, Houston) and vendor (ATCC; European Collection of Cell Culture).

For irradiation, cells at 80% confluence in 100 mm dishes were gamma irradiated (10Gy, 6 minutes) using a Mark 1 Irradiator (San Fernando, CA). Irradiated cells were immediately placed in fresh media and split at 2‐day intervals. At each splitting, conditioned media from irradiated and non‐irradiated cells were saved.

### 2.2 **SA‐**β**Gal staining**

PNT‐1α cells were analyzed for SA‐βGal activity using reported conditions.^18^ Briefly, PNT‐1α cells at 80%‐90% confluence were gamma irradiated, then changed to a fresh medium and after 5 days of culture, 1 × 10^5^of irradiated and non‐irradiated cells were seeded overnight in individual chambers of a polystyrene vessel tissue culture‐treated glass slide (1.7 cm^2^culture area, 4 wells, Falcon). Next day, cells were stained for SA‐βGal using a Kit (cat# 9860, Cell Signaling Tech., Danvers, MA).

### 2.3 Quantitative real‐time RT‐PCR and ELISA

Total RNAs, isolated by TRIZOL^®^, were converted to cDNAs using 5× iScript^TM^ cDNA Synthesis kit (Bio‐Rad, Hercules, CA) as before.^19^ Primers for various genes used in cDNA amplification are listed in Table 1. PCR amplification was done in the presence of iTaq^TM^SYBR^®^Green Supermix with ROX (Bio‐Rad) using a CFX384 Touch^TM^ Real‐time PCR detection system (Bio‐Rad).

**Table 1:** QRT‐PCR Primer sequences

IL‐1α	Forward	5′‐CGCCAATGACTCAGAGGAAGA‐
	Reverse	5′‐ AGGGCGTCATTCAGGATGAA‐
IL‐1β	Forward	5′ GCACGATGCACCTGTACGAT‐
	Reverse	5′ CACCAAGCTTTTTTGCTGTGAGT‐
IL‐6	Forward	5′ ATGAACTCCTTCTCCACAAGCGC‐
	Reverse	5′ GAAGAGCCCTCAGGCTGGACTG‐
IL‐8	Forward	5′‐CTTGGCAGCCTTCCTGATTT‐
	Reverse	5′‐TTCTTTAGCACTCCTTGGCAAAA‐
IL‐18	Forward	5′ ATGGCTGCTGAACCAGTAGAAG‐
	Reverse	5′ CAGCCATACCTCTAGGCTGGC‐
TNF‐α	Forward	5′ TCTTCTCGAACCCCGAGTGA‐
	Reverse	5′ ATGAGGTACAGGCCCTCTGA‐
CXCL12	Forward	5′ TGCCAGAGCCAACGTCAAG‐
	Reverse	5′ CAGCCGGGCTACAATCTGAA‐
MMP‐1	Forward	5' GGGCTTGAAGCTGCTTACGAATT‐
	Reversed	5' CAGCATCGATATGCTTCACAGTTCT‐
MMP‐3	Forward	5' GAGGCTGATATAATGATCTC ‐
	Reversed	5' TAAATTGGTCCCTGTTGTAT‐
MMP‐10	Forward	5′ GTTCTGGGCCATCAGAGGAAATG‐
	Reverse	5′ TCCTTGTCAGAAACAGCTGCATC‐
IGFBP‐2	Forward	5′ GCCCTCTGGAGCACCTCTACT‐
	Reverse	5′ CATCTTGCACTGTTTGAGGTTGTAC‐
IGFBP3	Forward	5′ GTCCAAGCGGGAGACAGAATAT‐3′
	Reverse	5′ CCTGGGACTCAGCACATTGA‐3′
GM‐CSF	Forward	5′CACTGCTGCTGAGATGAATGAAA‐
	Reverse	5′GTCTGTAGGCAGGTCGGCTC‐
β‐actin	Forward	5′CGTACCACTGGCATCGTGAT‐
	Reversed	5′GTGTTGGCGTACAGGTCTTT‐

For ELISA, GM‐CSF secreted in the cell culture media was quantified using a human GM‐CSF Quantikine ELISA kit (cat # DGM00, R&D Systems, Minneapolis, MN). Cells (±IR) were cultured for 9 days and media collected on day 9 was used for ELISA. Quantification was done on four biological replicates, with each sample assayed in triplicate. ELISA on a microplate reader was done using vendor‐instructed conditions.

### 2.4 Conditioned media, cell proliferation, pathway activation

Cells in 12‐well plates at 4 × 10^4^cells per well were cultured overnight in regular media and then placed in conditioned media (diluted 1:1 with fresh media) collected from day 6 or day 9 culture of irradiated or non‐irradiated cells. At 72‐hour post‐culture, viable cells were counted using an automated Countess^®^ Cell Counter (Invitrogen, CA). Average values from triplicate wells were calculated. For pathway activation assay, cells at 72 hours post‐culture were analyzed by Western blotting for levels of AKT/phospho‐AKT, ERK/phospho‐ERK, and STAT5/phospho‐STAT5. Western blot assay was performed on two biological replicates.

### 2.5 Western blotting, immunostaining of cells and prostate specimens

Cells lysates were prepared in RIPA buffer containing protease inhibitors as before^19^; protein amounts were quantified by Bradford assay. Lysates, cleared of debris, were analyzed by 10% SDS‐PAGE and Western blotting. Signals on x‐ray films were visualized using enhanced chemiluminescence (ECL). Size markers informed the size for each band. To analyze non‐phosphorylated vs phosphorylated signaling proteins, lysates at equal protein amounts were run in duplicate on the same gel and each half of the membrane was incubated with primary antibody specific to a phospho or non‐phospho signaling protein. X‐ray films were scanned with a Gel Doc EZ Imager (Bio Rad) using x‐ray film parameters in the Image Lab program of Gel Doc EZ.

Immunocytochemical staining for p16 was done with anti‐p16 rabbit polyclonal antibody (Santacruz, sc‐467) and goat anti‐rabbit Alexa 594 (Cat# A11037) as the secondary antibody. Cell nuclei were stained with DAPI using VECTASHIELD^®^ Mounting Medium with DAPI (Vector Lab, Burlingame, CA).

Immunohistochemistry staining for p16/INK4a was performed on paraffin sections (5 mµ) of formalin‐fixed BPH and normal prostate specimens using anti‐p16 antibody (1:200 dilution). Staining was visualized by horseradish peroxidase‐conjugated anti‐rabbit IgG (Mach 2 Rabbit‐HRP polymer, Biocare Medical, Concord, CA) and diamino benzidine, as before.^20^ Non‐immune serum did not produce any background staining. BPH specimens were from the Tissue Bank at the University of Texas Health San Antonio Specimens were collected and archived under informed consents from patients and following an IRB‐approved protocol.

### 2.6 Microscopy

Fluorescently labeled cells were photographed at 4X using a Fluorescent Microscope (Zeiss AxioCam‐2) and images were processed with AxioVision 4.8 software (Zeiss). SA‐βGal‐stained cells were photographed at 4× with an inverted microscope (Nikon Eclipse TE2000‐U) under auto exposure, and images were processed using the software NIS‐Elements BR 3.2. IHC staining was visualized with an Olympus microscope (BX‐41 with DP71 cooled digital camera and BSW software). Images were captured at 10×, 20×, and 40×.

### 2.7 Antibodies

p16 (sc‐467) and β‐actin (sc‐47778) antibodies were from Santa Cruz Biotech. (Dallas, TX). Antibodies to ERK1/2, Cat # 4685; phospho‐ERK1 (Thr202/Tyr204), Cat # 4377; AKT, Cat# 9272; phospho‐AKT (Ser473), Cat # 4060; phospho‐AKT (Thr308), Cat # 9275 were from Cell Signaling Tech. (Danvers, MA). Antibodies to STAT5, Cat # ab126832 and phospho‐STAT5 (Tyr 694), Cat # ab32364 were from Abcam (Cambridge. MA). Antibody specificities are ensured by the vendor‐provided results on websites.

### 2.8 Statistics

Data from two groups were compared using the two‐tailed *t* test. All *P* values are two sided. Results were considered significant at *P* ≤ 0.05. Microsoft Excel was used for statistical analysis.

## 3 RESULTS

### 3.1 Senescence of irradiated prostate epithelial and stromal cells

**Figure 1:**
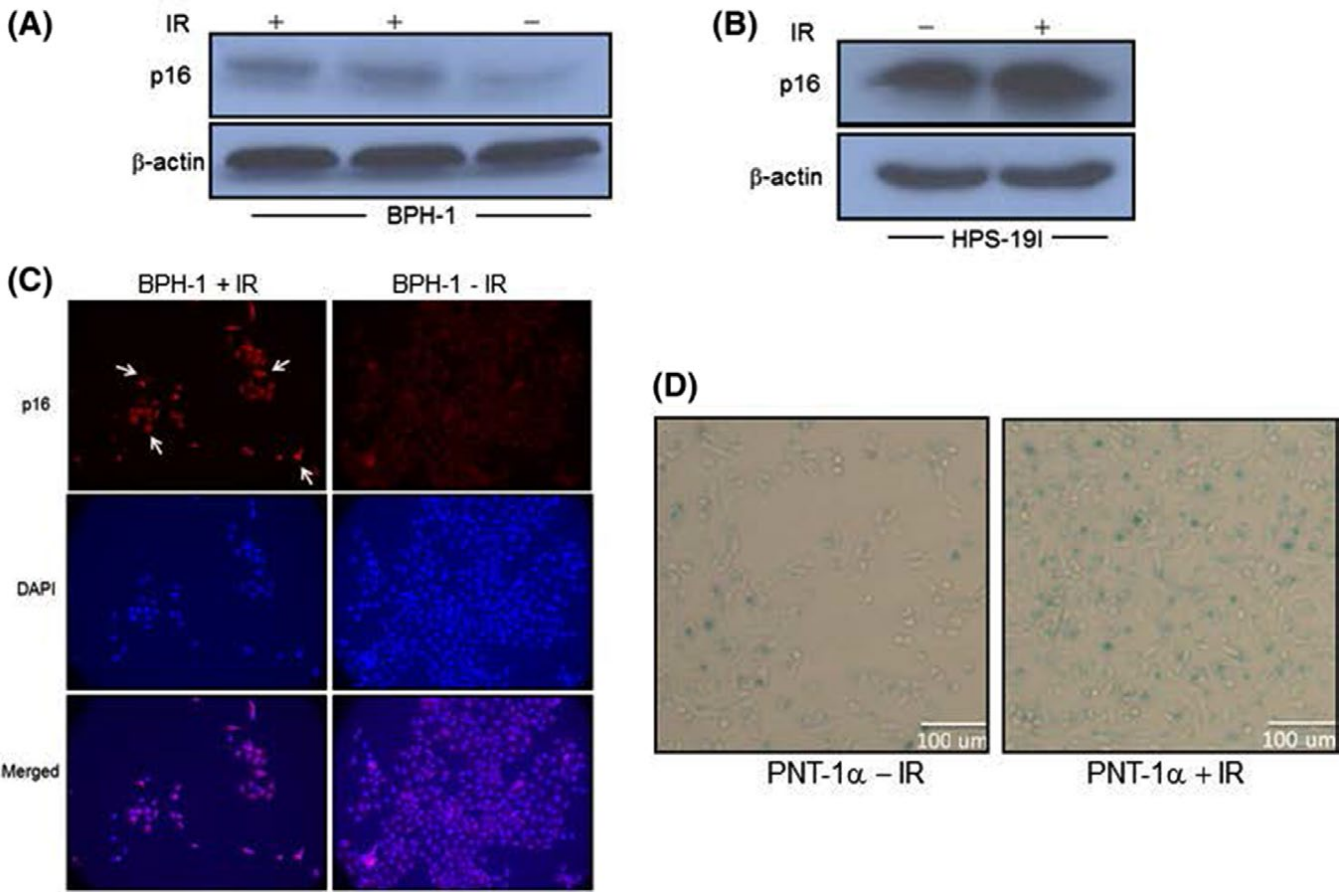
Cellular senescence in gamma‐irradiated epithelial and stromal prostate cells. A and B, Western blot of p16/INK4a levels in the lysates of BPH‐1 epithelial (A) and HPS‐19I stromal (B) cells. Cells with/without exposure to ionizing radiation (IR) were analyzed. Molecular size of protein bands was deduced from size markers. C, Immunofluorescent detection of p16‐expressing BPH‐1 cells with/without irradiation. Cells expressing p16 are shown by arrows. DAPI‐stained fluorescent nuclei of cells are shown (middle panel). Bottom panel shows merged images of p16‐expressing cells and corresponding DAPI‐stained nuclei. D, Senescence‐associated β‐galactosidase (SA‐βGal) in gamma‐irradiated PNT‐1α epithelial cells. Cells expressing SA‐βGal activity cleaved the chromogenic substrate X‐Gal at pH 6.0 to an insoluble blue‐colored product. A and B are representative results of two biological replicates. C and D are representatives of two biological replicates

DNA damage from exposure to IR from a gamma irradiator led to cellular senescence in BPH‐1 epithelial and HPS‐19I stromal prostate cells, evidenced by increased p16 levels in irradiated cells (Figure 1A,B). Immunocytochemical staining further demonstrated a markedly higher proportion of p16‐positive cells within the irradiated BPH‐1 cell population (Figure 1C). Increased cell death from DNA damage accounts for the smaller number of DAPI‐stained nuclei for IR‐exposed cell population compared to the untreated population (Figure 1C, middle panels). Almost all cells in the irradiated group, however, were p16‐positive. By contrast, most cells in the non‐irradiated group either lacked p16 or stained very weakly for p16 (Figure 1C, top panel). Irradiated HPS‐19I cells also had increased p16 levels (Figure 1B). High basal p16 expression in non‐irradiated HPS‐19I cells is likely due to replicative senescence for a significant fraction of primary HPS‐19I cells caused by repeated passaging of these cells since their isolation from the normal human prostate.^17^

Senescence‐associated β‐galactosidase activity in irradiated PNT‐1α epithelial cells was further evidence that ionizing radiation induced cellular senescence. A greater number of irradiated PNT‐1α cells expressed SA‐βGal activity than non‐irradiated cells (Figure 1D). At pH 6.0, SA‐βgal cleaves its chromogenic substrate 5‐bromo‐4‐chloro‐3‐indolyl β‐D‐galactopyranoside (X‐gal) to an insoluble blue compound. This activity differs from lysosomal β‐galactosidase, which is active at an acidic pH.

### 3.2 SASP of irradiated prostate cells

**Figure 2:**
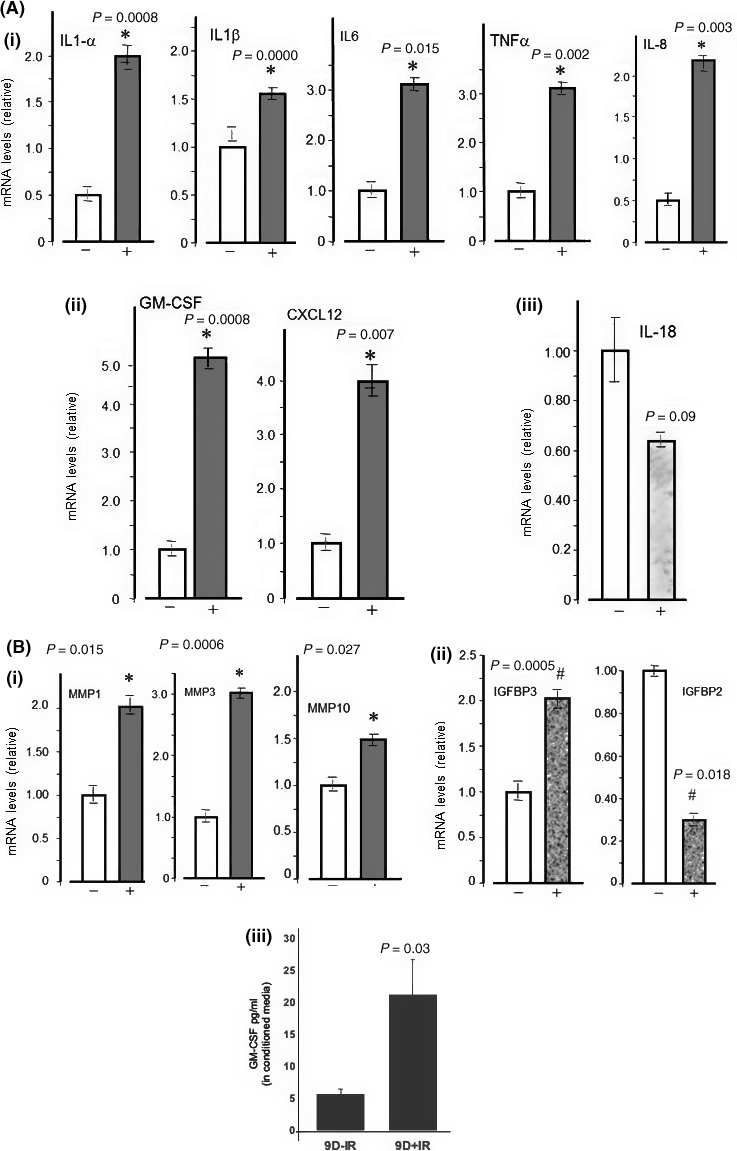
Induction of SASP components in irradiated BPH‐1 cells. A and B, qRT‐PCR assay of mRNAs for cytokines, growth factors, and proteases. Results were normalized to β‐actin mRNAs. The forward and reverse primer set of each test gene is described in Table 1. Each bar shows the average of three biological replicates, each conducted in duplicate. Data represent mean ± SEM. Significance is at *P* < 0.05. C, ELISA of conditioned media from 9‐day culture of BPH‐1 cells (±IR). Assay was done in the linear range of the analyte concentration, determined from the standard curve of the assay of recombinant human GM‐CSF. Each bar graph represents average ±SEM from three biological replicates. Each assay was done in triplicate

Ionizing radiation‐exposed BPH‐1 cells acquired a SASP, evidenced by mRNA induction for a number of known SASP components such as the pro‐inflammatory cytokines IL1‐α, IL‐1β, IL‐6, TNF‐α, IL‐8; the CXC‐motif chemokine ligand CXCL12; the growth‐promoting factors GM‐CSF, IGFBP3; and the matrix metalloproteases MMP1, MMP3, and MMP10 (Figure 2A, 2B). ELISA also showed ~4‐fold higher GM‐CSF protein levels in the culture media from IR‐exposed BPH‐1 cells (Figure 2C). These results are consistent with reports that senescent cells with persistent DNA damage or replicative exhaustion secrete cytokines, growth factors, proteases, and other factors that enhance cell proliferation through autocrine and paracrine activities.^7^ Increased inflammatory characteristics of irradiated cells conform to previous reports that GM‐CSF, IL‐1α, and IL‐8 levels are significantly higher in BPH tissue than transition‐zone normal prostate, and in late‐passage prostate epithelial cells than early‐passage cells.^6^ The pro‐inflammatory cytokine IL‐18 and IGF1‐binding IGFBP2 were reduced in irradiated BPH‐1 cells (Figure 2A‐iii; B‐ii). The significance of these reductions remains to be determined.

### 3.3 Stimulation of cell proliferation by SASP components

**Figure 3:**
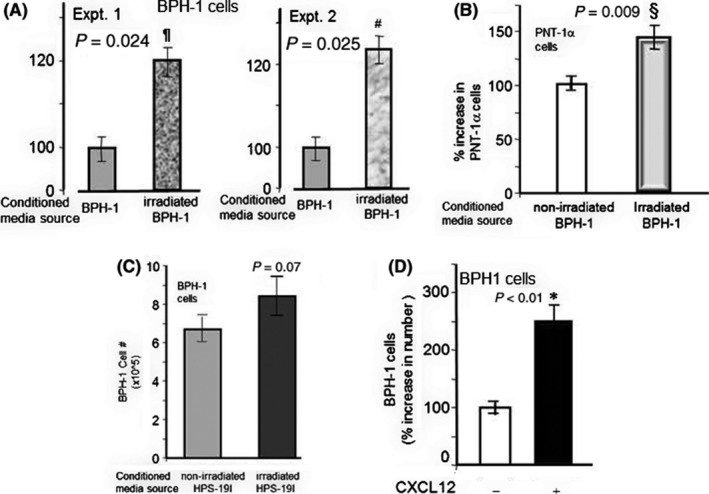
Effects of conditioned media of irradiated cells on cell proliferation. Sources of conditioned media are shown. Non‐irradiated cells were cultured in the presence/absence of conditioned media from irradiated BPH‐1 or irradiated HPS‐19I cells. A, Percent increase of BPH‐1 cells; two independent experiments, each conducted in triplicate. B, Percent increase of PNT‐1α cells—results produced from two biological replicates, each done in triplicate. C, BPH‐1 cell number after culture for 72 h in the presence/absence of conditioned media of irradiated HPS‐19I cells. The result for each is from assays in triplicate. D, BPH‐1 cells with/without stimulation by recombinant CXCL12. Bar graphs are averages of duplicate assay. *P* values are shown

BPH‐1 and PNT‐1α cells proliferated more rapidly in the presence of the conditioned media from irradiated BPH‐1 cells, indicating that the observed proliferation escalation was caused by factors secreted from IR‐exposed BPH‐1 cells (Figure 3A,B). Since senescence is induced with a slow kinetics, conditioned media from cells cultured for 9 days post‐irradiation was assessed for SASP effects on cell proliferation. Secreted factors from irradiated BPH‐1 cells significantly increased BPH‐1 proliferation in two independent assays (*P* = 0.024, *P* = 0.025) (Figure 3A). They also increased PNT‐1α epithelial cell proliferation (*P* = 0.009) (Figure 3B).

Factors secreted from irradiated HPS‐19I stromal cells also enhanced BPH‐1 proliferation, although the increase did not reach statistical significance (*P* = 0.07) (Figure 3C). It is likely that a higher basal SASP in the non‐irradiated HPS‐19I population, which was evident from elevated p16 expression (Figure 1B), diminished the difference in the rate of BPH‐1 cell proliferation under the two culture conditions, which contributed to a *P* value of 0.07. Recombinant CXCL12, which is a prominent SASP component, increased the BPH‐1 cell number by 2.5‐fold at 72 hours post‐culture (Figure 3D). We conclude that SASP in irradiated epithelial or stromal cells can cause prostate epithelial cells to proliferate more rapidly.

### 3.4 Activation of survival and growth‐promoting signals in a SASP environment

**Figure 4:**
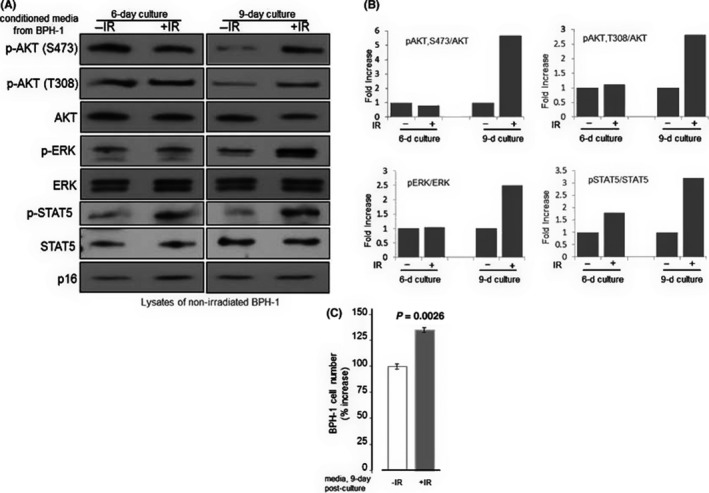
Activation of AKT, ERK, STAT5 in SASP‐exposed BPH‐1 cells. Non‐irradiated BPH‐1 cells were incubated for 72 hours with conditioned media collected at 6‐day and 9‐day cultures of non‐irradiated or irradiated BPH‐1 cells. A, Western blotting of cell lysates for phospho‐AKT, phospho‐ERK1/2, and phospho‐STAT5 and corresponding non‐phospho forms. Size markers informed molecular weights of the bands. Western blots for lysates from a second batch showed similar results. B, Quantification of the fold activation of signaling molecules. C, Proliferation stimulation of BPH‐1 cells by the conditioned media from the 9‐day culture of irradiated cells. The same 9‐day conditioned media was used for incubation of non‐irradiated BPH‐1 cells and subsequent Western blotting shown in Figure 4A

BPH‐1 cells, upon culture for 72 hours with the conditioned media from a 9‐day culture of irradiated BPH‐1 cells, showed elevated phospho‐AKT at threonine‐308 and serine‐473, and elevated phospho‐ERK1 at threonine‐202/tyrosine‐204, indicating increased AKT and ERK activities (Figure 4A). Total AKT and ERK1/2 levels did not change. Interestingly, elevated phospho‐STAT5 levels, indicative of increased STAT5 activity, were detected in cells exposed to the conditioned media from both 6‐day and 9‐day cultures (Figure 4A, bottom panels). The p16 levels were similar between non‐irradiated and irradiated cells. Image quantification of the phospho form of each signaling molecule, normalized to the corresponding non‐phospho form, showed 2.5‐ to 5‐fold activation (Figure 4B). Conditioned media from the 9‐day culture of irradiated BPH‐1 cells that caused activation of AKT, ERK1/2, and STAT5 (Figure 4A), significantly stimulated proliferation of BPH‐1 cell (Figure 4C).

Since secretions from the 6‐day culture enhanced STAT5 activation when changes in AKT and ERK1/2 phosphorylation were not detected, it is likely that the STAT5 response is more sensitive to the SASP components of irradiated cells. In view of a role for STAT5 in stimulating cell proliferation due to cyclin D1 induction,^21^ and roles of AKT and ERK1/2 in promoting proliferation, growth, and survival of cells,^22^, ^23^ we conclude that all three signaling pathways play roles in enhancing BPH‐1 cell growth and proliferation in the presence of SASP‐derived secreted factors.

### 3.5 Expression of p16/INK4a in BPH tissue

**Figure 5:**
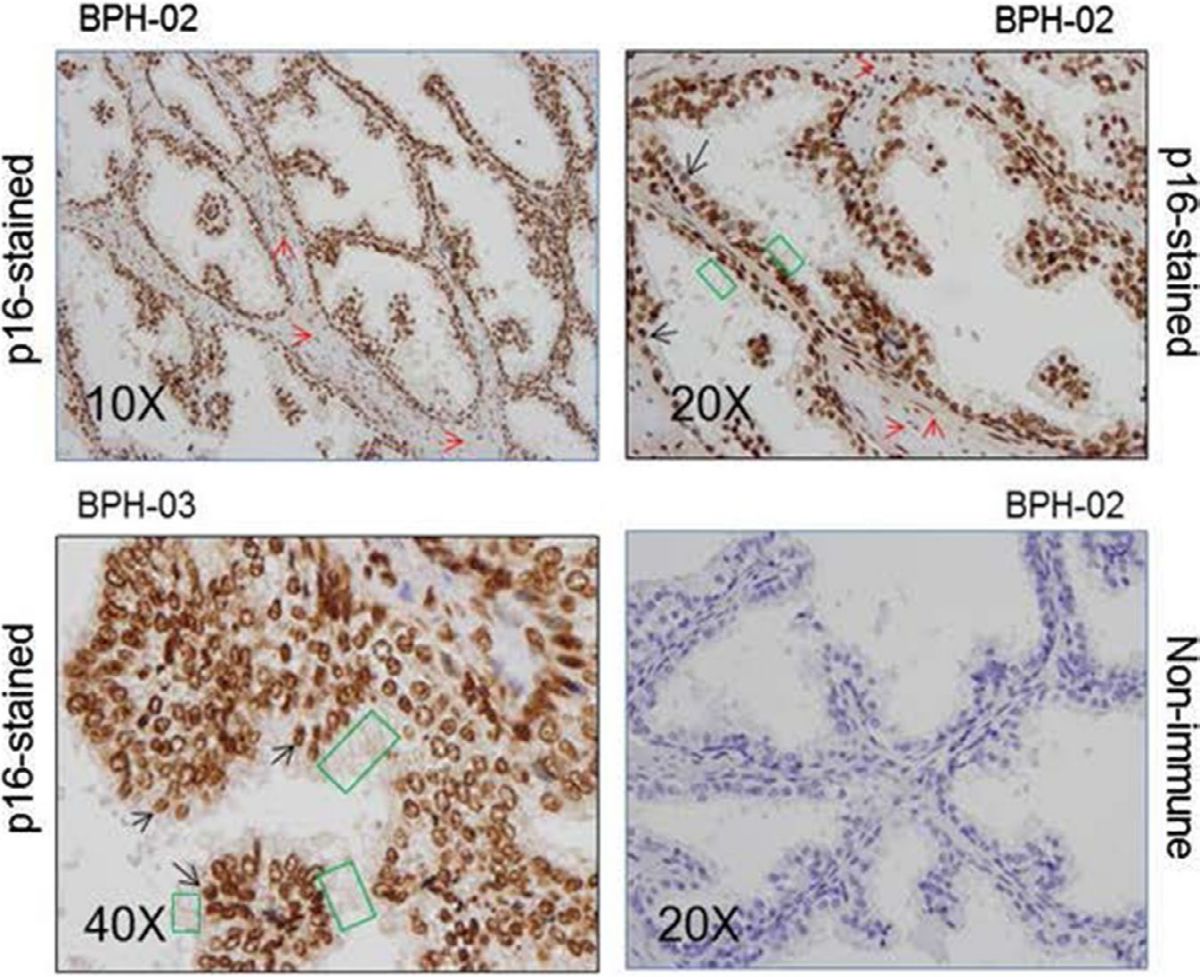
p16/INK4a expression in human BPH specimens. Immunohistochemical staining of BPH tissue from two patients—BPH‐02 and BPH‐03. Specificity for p16 staining is shown by the lack of staining with non‐immune rabbit anti‐serum. Specimens were obtained from the UTHSA Tissue bank. Specimens were collected after informed consents and following an IRB‐approved protocol

Given our supposition that SASP of senescent prostate cells contributes to cellular hyperproliferation that culminates in aberrant glandular prostate growth, we examined BPH specimens for the expression of p16/INK4a, which is a biomarker for cellular senescence. IHC of formalin‐fixed samples from two patients (BPH‐002, BPH‐003) showed many p16‐positive epithelial cells (black arrows) and less frequently, p16‐positive stromal cells (red arrowheads) (Figure 5). IHC staining was specific, since non‐immune serum did not stain the tissue. Nuclear staining for p16 (black arrows) and its absence in the cytoplasm (green boxes) of the luminal epithelial cells are shown at 40× for BPH‐03 and at 20× for BPH‐02. These results confirm that p16‐expressing senescent cells are present abundantly in the BPH epithelium and less abundantly in the BPH stroma.

## 4 DISCUSSION

We employed DNA damage‐induced premature senescence of gamma‐irradiated human prostate cells as a model to investigate the impacts of SASP on prostate epithelial cell proliferation and on signal transducers that regulate cell growth, proliferation, and survival. Increased p16 and SA‐βGal expression in irradiated cells indicated senescent cell accumulation. Secreted factors in the conditioned media from irradiated cells caused significant proliferation enhancement for two non‐malignant prostate epithelial lines—BPH‐1 and PNT‐1α (*P* = 0.025 and *P* = 0.009, respectively). SASP in irradiated BPH‐1 cells was associated with activation of STAT5, AKT, and the ERK1/2 Map kinase, and induction of mRNAs for pro‐inflammatory cytokines (IL‐1α, IL‐1β, IL‐6, IL‐8; TNF‐α) and other known SASP components such as the chemokines CXCL12, GM‐CSF; the IGF‐binding protein IGFBP3; and metalloproteases (MMP1, MMP3, MMP10). Abundant p16 expression in the epithelium and stroma of clinical BPH indicated the presence of senescent cells in BPH tissue.

Figure 5, showing abundant p16 expression in the epithelial but not stromal tissue of BPH, is in keeping with an earlier report that only epithelial cells of BPH specimens expressed the proliferation markers Ki‐67 and PCNA, indicating that epithelial cell hyperstimulation is predominantly involved in BPH pathogenesis.^2^ Genotoxic stress that led to SA‐βGal induction in gamma‐irradiated PNT‐1α epithelial cells in our study (Figure 1D), may also partly account for cellular senescence in vivo in BPH tissue, since DNA base lesions from oxidative damage are more extensive in BPH tissue than normal prostate, and the anti‐oxidant enzymes superoxide dismutase and catalase were detected at reduced levels in BPH tissue.^24^ SASP components such as IL‐1α, IL‐8, and GM‐CSF, which were elevated in BPH tissue,^6^ were also induced in our irradiated prostate cell senescence model. IL‐8, a diagnostic marker of BPH, can activate the myofibroblast phenotype of BPH reactive stroma.^16^, ^25^ Furthermore, IL1‐α induced fibroblast growth factors (FGF‐2 and FGF‐7) and promoted benign prostate tumor growth in a mouse model, and FGF‐2 and FGF‐7 were elevated in BPH epithelium.^6^, ^26^ Above parallels between the characteristics of radiation‐exposed prostate epithelial cells in vitro and senescent epithelial cells in vivo in BPH suggest that radiation‐induced prematurely senescent prostate epithelial cells can be a platform for dissecting regulatory factors that promote BPH pathology.

The prevailing view on BPH pathogenesis is that the cumulative effects of low‐level chronic stimulation of prostate cells by inflammatory secretions from the reactive stroma and infiltrated inflammatory cells promote excessive cell proliferation in aging prostate.^27^ Chemokines such as CXCL12 trigger infiltration of inflammatory cells to the prostate periacinar microenvironment. Reactive stroma, on the other hand, is induced by epithelial cell‐secreted inflammatory cytokines such as IL‐8 (aka CXCL8).^27^ Of relevance are the findings of Macoska and colleagues, who reported that mRNA levels of several CXC‐type chemokine ligands including CXCL12 and the interleukins IL‐11 and IL‐33 are higher in the stromal fibroblasts/myofibroblasts of prostates from elderly donors than younger donors.^28^ Also, primary fibroblasts from aging prostates secreted higher levels of CXCL1, CXCL5, and CXCL6, and stromal and epithelial prostate cells proliferated faster in the presence of CXCL12 and other CXC‐type cytokines.^28^ Our results that CXCL12, an activating ligand for the CXCR4 receptor, was induced in irradiated BPH‐1 cells and recombinant CXCL12 markedly enhanced BPH‐1 cell proliferation lend credence to the notion that chemokine interaction with inflammatory signals and reactive stroma plays an important role in BPH etiology and progression.

We show that STAT5 was activated (reflected from elevated tyrosine‐694 phosphorylation) in BPH‐1 cells that were cultivated in the presence of the conditioned media of irradiated BPH‐1 cells. STAT5 activation is consequential to cytokine interaction with a cognate cell surface receptor, when phosphorylation of the receptor‐associated Janus kinase (JAK) initiates a phosphorylation cascade that leads to STAT5 phosphorylation, nuclear translocation, and its subsequent function as a transcription factor.^29^ Our result on STAT5 activation is consistent with several reported findings—(a) STAT5 was activated in BPH‐1 cells by the CCL5 cytokine secreted from co‐cultured MOLT‐3 cells (a T lymphocytic line)^30^; (b) Activated STAT5 induced cyclin D1, which led to faster G1→S cell cycle progression and enhanced BPH‐1 cell proliferation^30^; (c) STAT5 signaling promoted leukocyte infiltration into epithelial microenvironment^30^; (d) Finally, prostate epithelial cells proliferated more rapidly upon co‐culture with leukocytes.^31^

Our results that AKT and ERK1/2 were activated in SASP‐exposed BPH‐1 cells indicate involvement of pathways activated by these signaling molecules in the proliferation enhancement of BPH‐1 by SASP. AKT is a serine‐threonine kinase, which promotes cell survival by blocking apoptosis as a result of the phosphorylation of certain components of the cell death machinery and its upstream regulators. AKT promotes cell growth and proliferation by activating mTORC1 and downstream effectors that stimulate protein synthesis and lipogenesis.^22^, ^32^ Mitogen‐activated serine/threonine kinases ERK1 and ERK2 promote cell proliferation and survival although, under certain conditions, they may induce apoptosis.^23^, ^33^ We should note that ERK1/2 activation due to DNA damage induced cellular senescence was also observed for cisplatin‐treated melanoma cells.^34^ The longer culture time required to activate AKT and ERK compared to STAT5 (9 hours vs 6 hours, Figure 4) may be due to delayed accumulation of the factor(s) that direct AKT and ERK activation.

In summary, this study provides new evidence that activation of STAT5, AKT, and ERK1/ERK2 by SASP of senescent prostate cells is associated with enhanced proliferation of non‐senescent prostate cells. Downstream effectors of these signaling molecules that interact with SASP components such as cytokines, chemokines, and growth factors are potential targets for improved BPH therapy. Of note, removal of senescent cells in the bone microenvironment by senolytic drugs or by other targeting strategies improved the mass, strength, and microarchitecture of bone in a mouse model of age‐onset osteoporosis,^35^ and senolytic drugs are under clinical evaluation for efficacy against osteoporosis and other age‐related diseases.^35^, ^36^ Finally, we note that an luteinizing‐hormone releasing hormone (LHRH) antagonist (Cetrorelix), which brought down serum testosterone to castrate levels in a rat model of BPH, reduced prostate size and blunted the expression of pro‐inflammatory cytokines and growth factors.^37^ Accordingly, the suitability of LHRH antagonism in BPH therapy is currently being explored.^38^ We speculate that the androgen/androgen receptor (AR)‐regulated signaling network may be impacted by the SASP secretome, and genetic or pharmacologic targeting of downstream effectors of the AR pathway may further inform new avenues for controlling BPH pathogenesis.

## CONFLICT OF INTEREST

The authors have no financial conflicts of interest.

## AUTHOR CONTRIBUTIONS

S.J generated Figures 1, 2, 3, part of Figure 4, and contributed to the write‐up of Methods. C.S generated Figure 4, analyzed data. B.C planned the study, obtained IHC data in coordination with Pathology Core (Figure 5), analyzed all results, and wrote the manuscript. All authors reviewed the manuscript.
